# Risk of unnatural death following self-harm in South Africa: development and validation of multivariable prognostic models

**DOI:** 10.1136/bmjment-2025-302473

**Published:** 2026-06-25

**Authors:** Veronika Whitesell Skrivankova, Roxanne Pelteret, Stephan Rabie, Mpho Tlali, Naomi Folb, Eliane Rohner, Chido Chinogurei, Yann Ruffieux, Soraya Seedat, Mary-Ann Davies, Gary Maartens, John Joska, Andreas D Haas

**Affiliations:** 1 Institute of Social and Preventive Medicine, University of Bern, Bern, Switzerland; 2 HIV Mental Health Research Unit, Department of Psychiatry and Mental Health, University of Cape Town, Rondebosch, South Africa; 3 Neuroscience Institute, University of Cape Town, Rondebosch, South Africa; 4 Centre for Integrated Data and Epidemiological Research, University of Cape Town, Cape Town, South Africa; 5 Medscheme, Cape Town, South Africa; 6 Division of Clinical Pharmacology, Department of Medicine, University of Cape Town, Cape Town, South Africa; 7 Department of Psychiatry, Stellenbosch University, Stellenbosch, South Africa; 8 University Hospital of Psychiatry and Psychotherapy, University of Bern, Bern, Switzerland

**Keywords:** Emergency Services, Psychiatric, Mental Health Services, Psychiatry

## Abstract

**Background:**

Recurrent self-harm is common and is associated with an increased risk of unnatural death including suicide and fatal accidents. We developed and validated prognostic models to stratify individuals by risk of unnatural death after healthcare presentation for non-fatal self-harm to support clinical decision-making and targeted prevention when capacity to deliver evidence-based intervention is limited.

**Methods:**

We used insurance claims and vital registration data from 6846 South African medical insurance beneficiaries aged ≥10 years who were discharged alive after healthcare presentation for self-harm during 2011–2021 to develop and validate models predicting unnatural death after non-fatal self-harm. We fitted competing-risk regression models to predict unnatural death within 1–3 years after presentation and discharge. Variable selection was guided by the least absolute shrinkage and selection operator. We used bootstrapping for internal validation and estimated optimism-corrected concordance indices (C-index), calibration intercepts and slopes, and for risk stratification the proportions of unnatural deaths captured across predicted-risk groups. Final models included age, sex, encounter characteristics, prior psychotropic medication use and selected prior mental disorder diagnoses.

**Results:**

The final models achieved optimism-corrected C-indices of 0.74 at presentation and 0.75–0.76 at discharge and identified 86–88% of observed unnatural deaths within 2 years among the 40% of individuals with the highest predicted risk. Models showed little evidence of systematic miscalibration (optimism-corrected calibration intercepts −0.02 to −0.01), but some overfitting (optimism-corrected calibration slopes 0.84–0.92). Using the model with the highest optimism-corrected C-index, the simplified discharge model, the observed 2-year risk of unnatural death was 0.15% (95% CI 0.06% to 0.32%) among the 60% of individuals with the lowest predicted risk, comparable to the risk among 1 249 760 individuals without a self-harm history (0.14%, (95% CI 0.14% to 0.15%)).

**Conclusions:**

The prediction models effectively rank individuals accessing private sector care in South Africa by their probability of unnatural death following healthcare presentation for non-fatal self-harm.

**Clinical implications:**

The models are intended to support clinical decision-making and guide the prioritisation of individuals with predicted high risk for targeted interventions, supporting more efficient allocation of mental health care capacity, which is often limited in low- and middle-income settings.

**WHAT IS ALREADY KNOWN ON THIS TOPIC**

- Prognostic models can accurately predict recurrent suicidal behaviour after self-harm, but their clinical utility remains debated.
- Most existing models were developed in high-income settings, with few models developed for low- and middle-income countries, where they may support more efficient use of limited mental health capacity.

**WHAT THIS STUDY ADDS**

- We developed and internally validated prognostic models to stratify individuals by risk of unnatural death after healthcare presentation for non-fatal self-harm.
- The models demonstrated good discrimination, acceptable calibration and good risk stratification, separating individuals at strongly increased risk of unnatural death after self-harm from those at low risk comparable to individuals without a history of self-harm.

**HOW THIS STUDY MIGHT AFFECT RESEARCH, PRACTICE OR POLICY**

- This study suggests that prognostic models based on routinely available clinical and administrative data can support risk stratification after self-harm, informing disposition decisions and prioritisation for suicide-specific care when specialist capacity is constrained.

## Introduction

 Every year over 700 000 people die by suicide globally.^1^ Nearly 80% of suicides occur in low- and middle-income countries (LMICs), with the highest rates reported in the WHO African region.^1^ South Africa ranks among the countries with the highest age-standardised suicide rates in this region, estimated at 21.1 per 100 000 persons in 2021.^1^

Self-harm behaviours, including non-suicidal self-injury and suicide attempts, are also critical public health issues, causing substantial psychological distress, functional impairment and placing considerable strain on healthcare systems.^2^,^4^ In 2021, global self-harm incidence rates were 99 per 100 000 person years in females and 56 in males; in LMICs, the corresponding rates were 91 and 42 per 100 000.^5^ In a previous study of the same South African cohort used in this study, we documented substantially higher self-harm rates: 183 per 100 000 in females and 90 in males, with 90% of cases involving intentional self-poisoning.^6^

Self-harm behaviours are often recurrent and may escalate in severity over time. Many individuals who die by suicide present with self-harm in the months preceding death.^7 8^ Our previous work using this study’s cohort found the risk of subsequent unnatural death was considerably increased among individuals discharged after self-harm: sevenfold in males and nearly fivefold in females compared with those without self-harm history.^6^

Recommended interventions for individuals presenting with self-harm include psychiatric admission in case of immediate risk, treatment of underlying mental disorders and suicide-specific interventions such as safety planning, structured post-discharge follow-up and psychotherapeutic approaches aimed at building adaptive coping and problem-solving skills to manage future crises.^9 10^ While effective, these interventions depend on specialist capacity. In LMICs, shortages of trained providers limit their reach to everyone who could benefit,^11 12^ underscoring the need to allocate scarce mental health resources strategically by prioritising individuals at highest risk.

Accurately identifying individuals at high risk of suicide remains challenging, especially in LMICs with scarce mental health resources. Risk assessment methods range from unstructured clinical interviews to self-report scales and prognostic models.^13 14^ Clinical interviews are resource-intensive and perform poorly in predicting future self-harm.^13 14^ Similarly, self-report scales lack sufficient diagnostic accuracy.^15^ Advanced statistical and machine learning models offer a promising alternative by enabling structured, data-driven risk stratification,^14 16^ with some models demonstrating reasonable predictive accuracy.^16^

This study aimed to develop and internally validate prognostic models to estimate individual risk of unnatural death after self-harm in medically insured populations in South Africa. We developed four models for distinct use cases, each with a different set of candidate predictors. Risk scores derived from the models may assist emergency care providers in disposition decisions and may help disease management specialists and care providers prioritise individuals at highest mortality risk when demand for recommended evidence-based interventions (eg, suicide-specific brief interventions) exceeds available capacity.

## Methods

### Study design and participants

We conducted a cohort study using data from a South African medical insurance scheme (1 January 2011–30 June 2020) and an HIV programme (1 January 2011–26 January 2021), deterministically linked to national vital registration data from the National Population Register (1 January 2011–26 January 2021). From a source population of 1 256 623 individuals aged 10 years or older, with known sex, who could be linked to the vital registration system, we included individuals with a non-fatal self-harm encounter to develop and validate prognostic models (online supplemental figure 1). A non-fatal self-harm encounter was defined as any healthcare encounter for self-harm (International Classification of Diseases (ICD-10) codes X60–X84) where the individual did not die on the day of the encounter or before hospital discharge. To illustrate the absolute risk difference, we also included individuals without a prior self-harm encounter in an analysis estimating the cumulative incidence of unnatural death in a population without a self-harm history (online supplemental figure 1). Further details on the characteristics of the source population, self-harm incidence and management within this cohort have been reported elsewhere.^6^ Model development followed guidelines for clinical prediction models^17^ and was reported in accordance with the TRIPOD (Transparent Reporting of a multivariable prediction model for Individual Prognosis or Diagnosis) statement.^18^

### Data sources

Insurance data included sociodemographic characteristics, ICD-10 diagnoses from inpatient and outpatient claims, medication claims and laboratory test results. Vital registration data included date and cause of death (classified as natural, unnatural or unknown). The vital registration dataset did not include ICD-10 codes for the specific causes of death. Death registration in South Africa is 93% complete among adults and slightly lower among children.^19^

### Outcome

The outcome was unnatural death, defined as fatalities from external causes, including suicide and accidents, identified using ICD-10 codes V01–Y98. Outcomes were assessed within 3 years after the outpatient self-harm encounter and within 3 years after discharge for admissions for non-fatal self-harm.

### Predictors

We selected candidate predictors based on prior clinical and epidemiological knowledge of risk factors for self-harm and suicide. Selected factors included sex,^6^ age,^6^ mental disorder diagnoses,^20^ use of psychiatric medication,^21^ healthcare setting (outpatient care or hospital admission), encounter type (outpatient care, hospital admission of ≤3 days or hospital admission >3 days), encounter type^21^ and self-harm method at the index encounter.^6 22^ In an earlier version of the models, we also considered HIV status^23^ as a candidate predictor. HIV status was selected in most models and was associated with lower risk of unnatural death. This association is clinically implausible^23^ and likely spurious, reflecting strong confounding by race. In the current version of the models, we excluded HIV status from the set of candidate predictors, as this apparent protective association is unlikely to generalise across settings. Details on case definitions for each candidate predictor are shown in online supplemental table 1.

*Mental disorders* were grouped as organic mental disorders, substance use disorder, alcohol use disorder, drug use disorders, psychotic disorder, bipolar disorder, depression, anxiety disorder, post-traumatic stress disorder (PTSD), other anxiety disorders, personality disorder and other mental disorders. For individuals diagnosed with both depression and bipolar disorder, only the bipolar disorder diagnosis was considered, as bipolar disorder includes depressive episodes.

*Psychiatric medication* use was identified based on Anatomical Therapeutic Chemical (ATC) classification codes from medication claims, including antipsychotics, anxiolytics, antidepressants and drugs with proven anti-suicidal effect.

*Self-harm method* at index encounter was classified by lethality as highly lethal or less lethal, based on evidence that individuals using highly lethal methods at the index event are at greater risk of suicide^22^ and unnatural death.^6^ Highly lethal methods included hanging, gas poisoning, jumping from a height, firearms and drowning. Less lethal methods included drug or chemical poisoning, fire-related injuries, motor vehicle accidents, sharp or blunt object injuries, and other or unspecified methods.^24^

### Statistical analysis

We followed individuals from the date of their first observed self-harm outpatient encounter or, for hospitalised encounters, from the discharge date of the first observed self-harm admission. We did not consider repeated self-harm encounters, and each individual contributed only one index episode to the analysis. For individuals without a history of self-harm, follow-up began at the latest of medical scheme or HIV programme enrolment, their 10th birthday or 1 January 2011. For both groups, follow-up ended at the earliest of the following: database closure date, death or the maximum follow-up duration of 3 years. We modelled age as a continuous variable using a restricted cubic spline with 5 degrees of freedom, with knots at 25 and 35 years and boundary knots at 10 and 95 years.

We developed four models for predicting unnatural death for distinct use cases, each with a different set of candidate predictors. The *full presentation model* included the following candidate predictors: age, sex, healthcare setting, self-harm method and pre-index history of mental health diagnoses and psychiatric medication use, derived from each individual’s full available administrative records prior to the index encounter (online supplemental table 1). The *full discharge model* included the following candidate predictors: age, sex, encounter type, self-harm method and pre-index history of psychiatric medication use and mental disorder diagnoses, derived from each individual’s full available administrative records before the index admission, in addition to mental disorder diagnoses recorded during the index admission. The discharge model is intended to support decision-making at discharge, using all information available by discharge, including diagnoses made during the current admission. We also fitted a *simplified presentation model* and a *simplified discharge model* that excluded mental health diagnoses from the list of candidate predictors. These simplified models are intended for settings where mental health diagnoses are not routinely available (eg, the South African public sector).

We selected predictors using least absolute shrinkage and selection operator (LASSO) regression with 20-fold cross-validation, using the cross-validated concordance index (C-index) to select the penalty parameter. Predictor selection followed a two-stage approach. First, we screened binary predictors and retained those with at least two unnatural deaths within 2 years in both exposure groups. We applied the same minimum-event rule to interactions between sex and mental disorders, psychiatric medication and self-harm method, requiring at least two unnatural deaths within 2 years in each sex-by-exposure subgroup. Second, we fitted penalised LASSO models including all selected main effects and interaction terms. We selected predictors at the value of λ that maximised the cross-validated C-index. After selection, we enforced hierarchical structure by retaining the corresponding main effects for selected interaction terms and retained all levels of multilevel categorical predictors if any level was selected.

We then refitted the selected predictors in Fine-Gray subdistribution hazards models to estimate the cumulative incidence of unnatural death. We assessed model performance in terms of discrimination, calibration and risk stratification. Discrimination was quantified using the C-index for competing risks. Calibration was assessed graphically by comparing observed and predicted 1-year, 2-year and 3-year cumulative incidence across deciles of predicted risk using Aalen-Johansen estimates and numerically using 2-year calibration intercepts and slopes estimated on the complementary log-log scale with inverse probability of censoring weighting to account for censoring before 2 years. Risk stratification was assessed by quantifying the proportion of all unnatural deaths occurring within 1, 2 and 3 years that were captured within high-risk groups defined by increasing thresholds of predicted 2-year risk.

We used bootstrap internal validation with 1000 resamples to quantify optimism in discrimination (C-index), calibration intercept and slope, and risk stratification, assessed as the proportion of unnatural deaths captured within high-risk groups. In each bootstrap resample, we repeated the full model-development procedure, including predictor and interaction screening based on minimum-event rules, penalised LASSO selection using 20-fold cross-validation, enforcement of hierarchical selection rules, and refitting of the selected Fine-Gray model. We estimated optimism-corrected performance by subtracting the mean bootstrap optimism from the apparent estimates in the original dataset. We ranked predictors by the proportion of variance in the linear predictor (risk score) accounted for by each predictor term.

To illustrate absolute risk differences over time, we used the model with the highest optimism-corrected C-index, the simplified discharge model, to define high-risk groups as individuals in the top 10% and top 40% of predicted risk. For each group, we calculated Aalen-Johansen cumulative incidence functions for unnatural death, comparing them to the remainder of the self-harm cohort and to individuals without a prior non-fatal self-harm encounter. Further details are provided in online supplemental text 1.

## Results

### Characteristics of the study population

From a source population of 1 256 623 individuals, we included 6846 with a non-fatal self-harm healthcare encounter to develop and validate the prognostic models. A total of 1 249 760 individuals were included in the analysis of unnatural death in the population without prior non-fatal self-harm (online supplemental figure 1).

Among the 6846 individuals with a non-fatal self-harm encounter included in model development and validation, 4749 (69%) were female and 2097 (31%) were male. Most encounters were hospital admission of 3 days or fewer (66%) (table 1). The median age at the self-harm encounter was 31 years (IQR 21–41). Most encounters (98%) involved less lethal self-harm methods, most commonly poisoning; highly lethal methods were rare especially among females. Half of the individuals (51%) had received a diagnosis of a mental disorder prior to the self-harm encounter and 70% before discharge.

**Table 1:** Characteristics of model development and validation cohort

Characteristics	Female N=4749	Male N=2097	Total N=6846
Encounter type			
Outpatient	815 (17.2%)	561 (26.8%)	1376 (20.1%)
Admission >3 days	680 (14.3%)	306 (14.6%)	986 (14.4%)
Admission ≤3 days	3254 (68.5%)	1230 (58.7%)	4484 (65.5%)
Self-harm method			
Highly lethal	54 (1.1%)	82 (3.9%)	136 (2.0%)
Less lethal	4695 (98.9%)	2015 (96.1%)	6710 (98.0%)
Age at self-harm encounter, years			
Median (IQR)	30 (21, 40)	34 (23, 43)	31 (21, 41)
HIV status			
Negative	3670 (77.3%)	1695 (80.8%)	5365 (78.4%)
Positive	1079 (22.7%)	402 (19.2%)	1481 (21.6%)
Diagnoses received before self-harm encounter			
Any mental disorder	2446 (51.5%)	1046 (49.9%)	3492 (51.0%)
Organic mental disorders	53 (1.1%)	41 (2.0%)	94 (1.4%)
Alcohol use disorders	49 (1.0%)	80 (3.8%)	129 (1.9%)
Drug use disorders	49 (1.0%)	96 (4.6%)	145 (2.1%)
Psychotic disorders	62 (1.3%)	37 (1.8%)	99 (1.4%)
Bipolar disorder	467 (9.8%)	196 (9.3%)	663 (9.7%)
Major depression	1383 (29.1%)	529 (25.2%)	1912 (27.9%)
Anxiety disorders	1563 (32.9%)	597 (28.5%)	2160 (31.6%)
PTSD	152 (3.2%)	59 (2.8%)	211 (3.1%)
Other anxiety disorders	1516 (31.9%)	570 (27.2%)	2086 (30.5%)
Personality disorders	15 (0.3%)	11 (0.5%)	26 (0.4%)
Other mental disorders	420 (8.8%)	217 (10.3%)	637 (9.3%)
Diagnoses received up to discharge			
Any mental disorder	3402 (71.6%)	1419 (67.7%)	4821 (70.4%)
Organic mental disorders	81 (1.7%)	65 (3.1%)	146 (2.1%)
Alcohol use disorders	71 (1.5%)	131 (6.2%)	202 (3.0%)
Drug use disorders	86 (1.8%)	134 (6.4%)	220 (3.2%)
Psychotic disorders	82 (1.7%)	60 (2.9%)	142 (2.1%)
Bipolar disorder	597 (12.6%)	260 (12.4%)	857 (12.5%)
Major depression	2366 (49.8%)	878 (41.9%)	3244 (47.4%)
Anxiety disorders	1753 (36.9%)	681 (32.5%)	2434 (35.6%)
PTSD	172 (3.6%)	62 (3.0%)	234 (3.4%)
Other anxiety disorders	1700 (35.8%)	652 (31.1%)	2352 (34.4%)
Personality disorders	21 (0.4%)	17 (0.8%)	38 (0.6%)
Other mental disorders	437 (9.2%)	227 (10.8%)	664 (9.7%)
Drugs claimed before self-harm encounter			
Antipsychotics	1013 (21.3%)	413 (19.7%)	1426 (20.8%)
Antidepressants	1926 (40.6%)	718 (34.2%)	2644 (38.6%)
Anxiolytics	1812 (38.2%)	644 (30.7%)	2456 (35.9%)
Drugs with proven anti-suicidal effect	69 (1.5%)	23 (1.1%)	92 (1.3%)
Mortality censored at 3 years after self-harm	93 (2.0%)	111 (5.3%)	204 (3.0%)
Natural causes	64 (1.3%)	47 (2.2%)	111 (1.6%)
Unnatural causes	24 (0.5%)	56 (2.7%)	80 (1.2%)
Unknown	5 (0.1%)	8 (0.4%)	13 (0.2%)
Follow-up time in years, median (IQR)	3.00 (2.57, 3.00)	3.00 (2.39, 3.00)	3.00 (2.49, 3.00)

Data n (%), unless indicated otherwise.

PTSD, post-traumatic stress disorder.

### Unnatural death after non-fatal self-harm

At 3 years after the self-harm encounter, we observed 80 unnatural deaths (1.2%) over a median follow-up of 3.0 years (IQR 2.5–3.0) (table 1).

### Selected multivariable prognostic models

**Figure 1:**
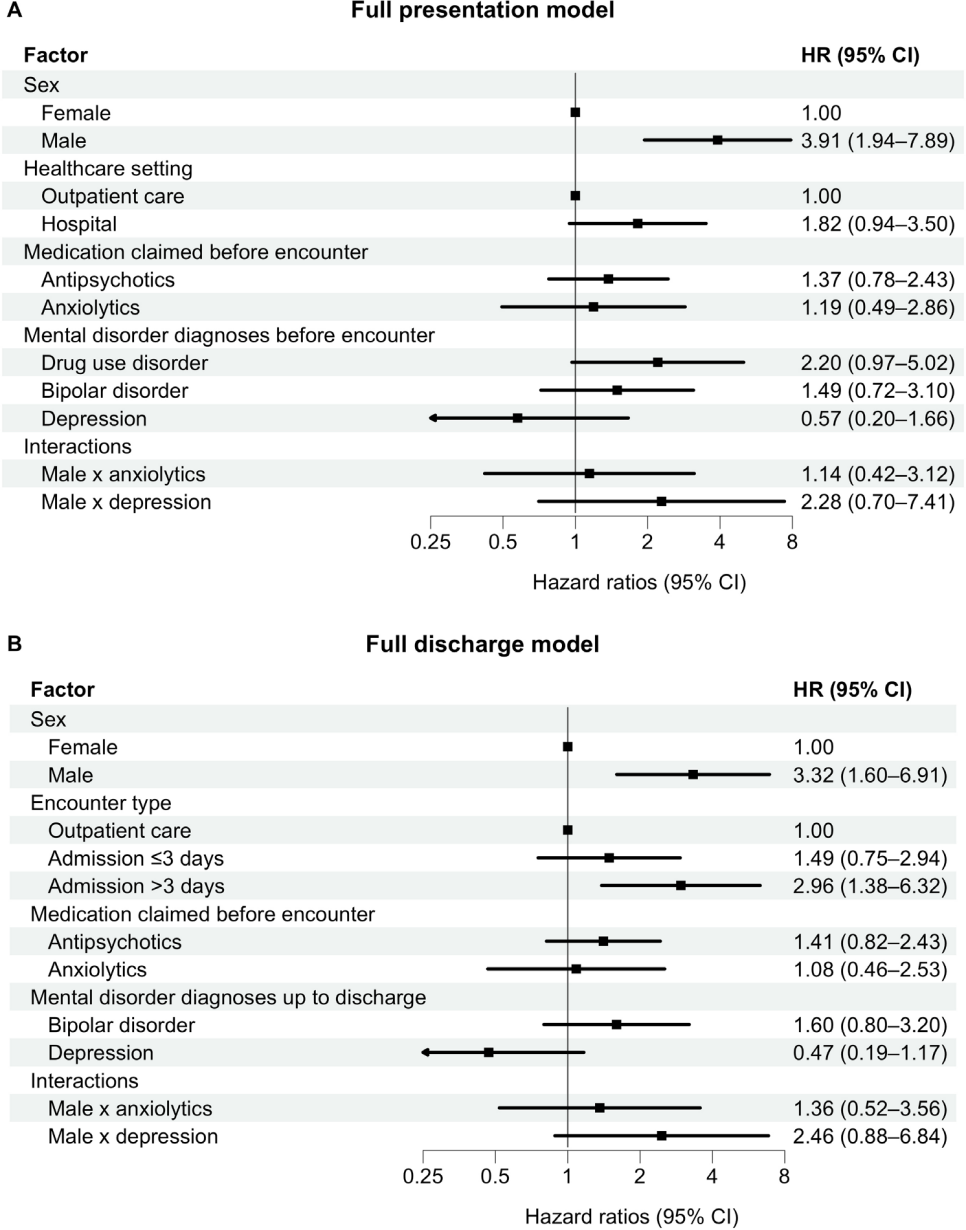
Adjusted HRs for categorical predictors in the final models. Estimates are subdistribution HRs with 95% CIs for unnatural death after non-fatal self-harm. (A) Results for categorical predictors included in the full multivariable presentation model. (B) Results for categorical predictors included in the full discharge model. For variables included in interaction terms, the reported HRs refer to the reference category of the interacting variable. Both models also included age, modelled as continuous predictor with restricted cubic splines. The association for age is shown in online supplemental figure 2.

The full presentation model with the highest cross-validated C-index included age, sex, healthcare setting, antipsychotic and anxiolytic use prior to the self-harm encounter, prior diagnoses of drug use disorder, bipolar disorder and depression, and interactions between sex and anxiolytic use and between sex and depression. The full discharge model with the highest C-index included age, sex, encounter type, antipsychotic and anxiolytic use before the self-harm encounter, diagnoses of drug use disorder and depression, and interactions between sex and anxiolytic use, and between sex and depression. Figure 1 and online supplemental figure 2 show adjusted HRs for predictors in the full models. The best-performing simplified presentation model included age, sex, healthcare setting, antipsychotic and anxiolytic use before the self-harm encounter, and interactions between sex and anxiolytic use. The best-performing simplified discharge model included age, sex, encounter type, and antipsychotic or anxiolytic use before the self-harm encounter (online supplemental figures 2 and 3). LASSO cross-validation performance across penalty parameters is shown in online supplemental figure 4.

Across all four models, sex and age contributed most to variation in the risk score. Sex accounted for 25–51% and age for 18–23% of the variance in the linear predictor. Healthcare setting or encounter type contributed 5–10%. Individual clinical predictors generally contributed less, although depression diagnosis accounted for 6% in the full presentation model and 12% in the full discharge model. Interaction terms made smaller contributions, with the sex by depression interaction accounting for 5–8% in the full models (online supplemental table 2).

### Model performance: discrimination, calibration

The four models showed similar performance across all indices, with optimism-corrected C-indices of 0.74 for the full presentation model, 0.75 for the full discharge model, 0.74 for the simplified presentation model, and 0.76 for the simplified discharge model (online supplemental table 3). Optimism-corrected calibration intercepts at 2 years ranged from −0.02 to −0.01 across models, suggesting little evidence of systematic overprediction or underprediction. Optimism-corrected calibration slopes ranged from 0.84 to 0.92, indicating some overfitting, with predicted risks that were somewhat too extreme (online supplemental table 3). Calibration plots showed good agreement between predicted and observed risks at 1, 2 and 3 years for all four models (online supplemental figures 5 and 6).

### Risk stratification performance

Table 2 and online supplemental figures 7 and 8 show the proportions of unnatural deaths captured within increasingly larger high-risk groups, defined by thresholds of predicted risk, at 1, 2 and 3 years for each of the four prognostic models. Optimism-corrected estimates indicated that, at 2 years, the 20% of individuals with the highest predicted risk captured 47% of unnatural deaths in the full presentation model, 57% in the full discharge model, 50% in the simplified presentation model and 51% in the simplified discharge model. Expanding the high-risk group to the top 40% captured 86%, 88%, 86% and 86% of unnatural deaths, respectively.

**Table 2:** Risk stratification trade-off between the population classified as high risk and unnatural deaths captured

Model	Prediction time point	Optimism-corrected capture of unnatural deaths (%)						
		Top 10%	Top 20%	Top 30%	Top 40%	Top 50%	Top 60%	Top 70%
Full presentation model	1 year	27.2	53.1	79.4	84.2	92.0	94.6	94.9
	2 years	27.9	47.3	74.8	85.9	91.8	95.6	95.9
	3 years	28.9	46.3	69.3	78.5	83.8	89.0	90.8
Full discharge model	1 year	31.0	62.9	81.4	86.5	92.0	92.3	92.7
	2 years	28.5	57.2	75.9	87.6	91.9	92.3	94.4
	3 years	30.1	55.5	69.7	82.3	86.7	88.1	92.3
Simplified presentation model	1 year	32.1	52.3	76.3	81.9	86.8	92.0	97.1
	2 years	27.7	49.6	72.8	85.8	89.8	93.8	97.6
	3 years	29.2	48.2	68.1	78.9	81.7	88.3	91.1
Simplified discharge model	1 year	48.1	56.6	81.1	84.8	85.1	87.4	97.2
	2 years	42.1	51.0	74.7	86.1	88.7	90.7	97.7
	3 years	40.8	50.3	70.6	80.4	83.6	86.3	92.7

Values show the proportion of all unnatural deaths occurring within 1, 2 and 3 years that were captured within increasingly larger high-risk groups defined by the top 10% to 70% of the cohort with the highest predicted 2-year risk. Results are shown for the full presentation, full discharge, transportable presentation, and transportable discharge models. Optimism-corrected estimates were obtained from bootstrap internal validation of the full model development pipeline.

### Absolute risk in risk groups

**Figure 2:**
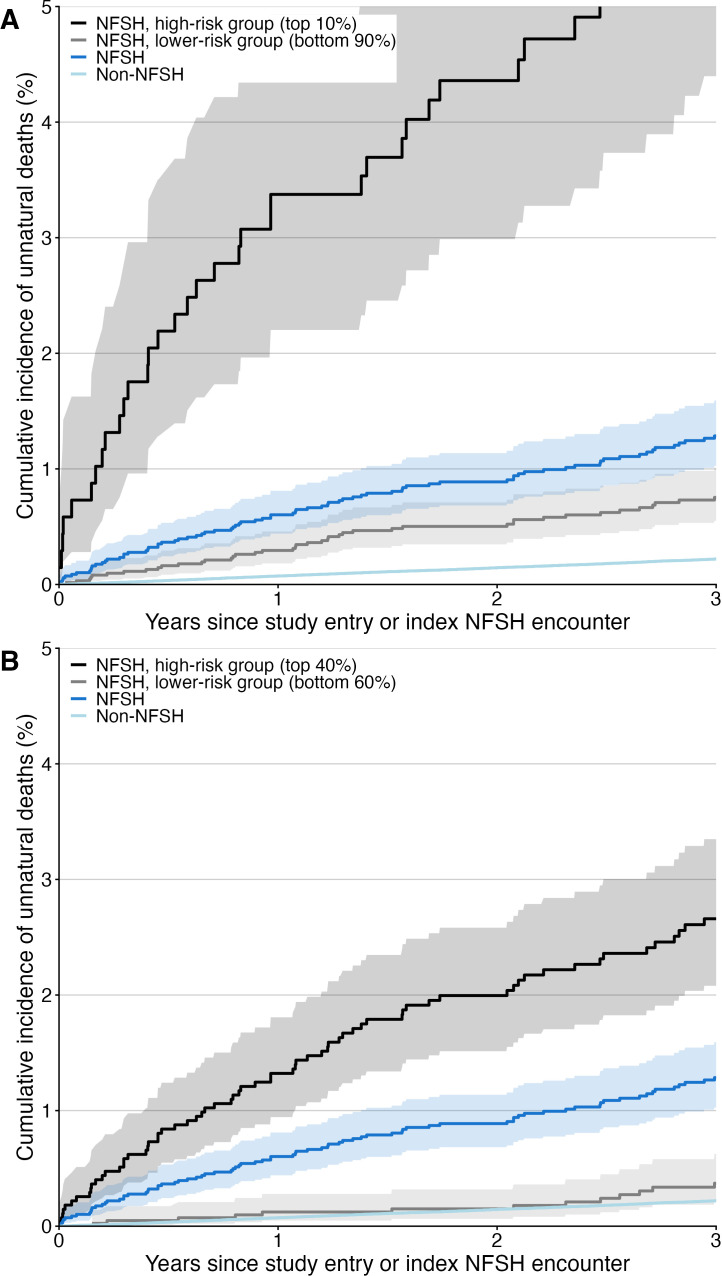
Cumulative incidence of unnatural death after non-fatal self-harm (NFSH) in high-risk and low-risk groups at varying thresholds, compared with individuals without prior self-harm. The figure shows cumulative incidence of unnatural death with 95% CIs after NFSH among high-risk groups, defined as the top 10% (A) and top 40% (B) by predicted risk, compared with the corresponding lower-risk groups (bottom 90% in A and bottom 60% in B) and individuals without a prior NFSH encounter. Predictions are based on the presentation model.

Figure 2 shows the cumulative incidence of unnatural death after non-fatal self-harm for high-risk groups defined using the simplified discharge model as the top 10% and 40% of individuals with the highest predicted risk, compared with the corresponding lower-risk groups and individuals without a prior non-fatal self-harm encounter. At 2 years after the self-harm encounter, the cumulative incidence of unnatural death was 4.36% (95% CI 2.99% to 6.11%) in the top 10% risk group, compared with 0.50% (95% CI 0.35% to 0.71%) in the bottom 90% group (figure 2A). In the top 40% risk group, the cumulative incidence was 2.00% (95% CI 1.51% to 2.58%), compared with 0.15% (95% CI 0.06% to 0.32%) in the bottom 60% group and 0.14% (95% CI 0.14% to 0.15%) among individuals without prior self-harm (figure 2B). Corresponding plots based on the discharge model are shown in online supplemental figure 9.

## Discussion

In South Africa’s private healthcare sector, self-harm rates are almost twice the global average.^5 6^ Individuals presenting with self-harm have a fivefold to sevenfold higher risk of subsequent suicide and other unnatural deaths.^6^ These findings underscore the need to implement evidence-based interventions to reduce recurrent suicidal behaviour and prevent suicide in this high-risk group.^9^ However, managing patients after self-harm poses substantial challenges. Frontline healthcare workers often face difficult disposition decisions about who can be managed safely as an outpatient and who requires inpatient care. In addition, health systems in LMICs must allocate scarce mental health resources,^11 12^ including limited psychiatric bed capacity and limited capacity to deliver specialised services such as suicide-specific interventions.

To support disposition decisions and resource allocation, we developed and internally validated prognostic models to estimate the risk of unnatural death at first healthcare presentation after non-fatal self-harm (presentation model) and after discharge following self-harm-related hospital admission (disposition model). All four models showed similar optimism-corrected discrimination, with C-indices ranging from 0.74 to 0.76. Calibration intercepts were close to 0, suggesting little evidence of systematic overprediction or underprediction. However, calibration slopes below 1 indicated some overfitting, with predicted risks that were somewhat too extreme, particularly at the lower and higher ends of the risk distribution. The models should therefore be interpreted primarily as tools for risk ranking and stratification rather than precise estimation of absolute risk. Risk stratification remained clinically relevant: using optimism-corrected estimates, the 40% of individuals with the highest predicted risk captured 86–88% of observed unnatural deaths within 2 years. By contrast, the corresponding 60% of individuals with the lowest predicted risk had a low observed risk of unnatural death, comparable to individuals without prior self-harm. If confirmed in external validation, the models could be used to support clinical decision-making by identifying individuals at low risk of unnatural death who may be managed safely as outpatients and by prioritising individuals at elevated risk for more detailed assessment and suicide-specific interventions when demand exceeds capacity.

The predictors retained in our model are clinically recognisable and may already be considered informally by clinicians when evaluating patients after self-harm. Yet reliance on unstructured clinical judgement alone has long been shown to poorly predict future suicidal behaviour, performing only slightly better than chance.^13 14^ Such assessments are time-consuming, prone to bias, and inconsistent across providers, with outcomes influenced by setting, timing, and workload. These challenges are compounded by the fact that most self-harm presentations in South Africa occur after hours, when triage decisions often fall to junior or rotating doctors with limited training and expertise in suicide prevention.^25^ In these circumstances, clinicians may understandably err on the side of caution, generating referral and admission volumes that may exceed capacity and place additional pressure on services.

Our models address some of these challenges by formalising familiar predictors into structured, probability-based risk estimates with known diagnostic accuracy. These model-informed estimates could help standardise decision-making across providers in various healthcare settings and reduce some of the psychological burden of making high-stakes triage decisions under pressure with limited support. Importantly, the models are intended to complement, not replace, clinical judgement. Clinicians retain discretion to override model-informed recommendations when additional risk factors are present or when clinical circumstances warrant a different course. For example, a low predicted risk may support outpatient management, but clinicians may still admit a patient if they identify relevant risk factors that are not included in the risk score (eg, acute intoxication, ongoing suicidal intent, or limited social support) or other indications for inpatient care. Importantly, clinicians should not use the individual model coefficients presented in this study to informally estimate risk, because these coefficients come from multivariable prognostic models and do not represent independent or causal effects of individual risk factors. Risk estimation should instead rely on prognostic scores from the validated models.

Although the models use only five to eight predictors, implementation depends on access to longitudinal clinical information. In the private sector, emergency department clinicians typically do not have real-time access to patients’ claims data, which limits immediate point-of-care use of the presentation model. Clinicians could potentially obtain predictor information by patient report, but predictors measured in administrative claims data may not perform equivalently when measured by self-report because measurement error and reporting biases differ by data source. Any translation to self-reported predictors would therefore require further validation and may require recalibration. A more feasible near-term pathway in the private sector is implementation within medical scheme disease management workflows, where disease management specialists can apply the discharge model to routinely collected claims data to generate automated risk scores and alerts. This approach could support proactive outreach to providers or patients and allocation of care in proportion to predicted risk. In public sector settings with advanced integrated health information systems, such as the Western Cape, similar models could be operationalised directly at the point of care because clinicians can access longitudinal records through platforms such as the Single Patient Viewer provided by the Western Cape Provincial Health Data Centre.

By producing continuous risk estimates, our models allow prioritisation thresholds to be tailored to clinical need and system capacity. This approach offers advantages over earlier rule-based instruments, such as the Manchester Self-Harm Rule (MSHR)^26^ and ReACT Self-Harm Rule,^27^ which reduced suicide risk to binary classifications. In contrast, our outputs support stratification by degree of risk, enable proportionate clinical responses and can be adapted to local service capacity.^14^

Existing prognostic models for outcomes after self-harm have been developed almost exclusively in high-income countries.^14 16^ The recently developed Oxford Suicide Assessment Tool for Self-Harm (OxSATS) is a well-performing example from a high-income context. Derived from Swedish registry data, the model achieved good discrimination (C-index 0.77) in external validation but incorporated a broad range of sociodemographic and psychiatric predictors typically available in high-income settings.^21^ By contrast, our models achieved similar discrimination in internal validation using a parsimonious set of five to eight routinely collected clinical and administrative predictors in South Africa, which improves feasibility in LMIC settings where registry infrastructure and diagnostic ascertainment are limited. We additionally developed simplified versions of the models that excluded mental health diagnoses, which are captured incompletely in many South African settings, including the public sector. These simplified models showed similar discrimination risk stratification performance, demonstrating that models could be applied in settings without available mental health diagnoses. Together, these findings highlight the importance of tailoring prediction models to local data availability and service structures.^17 28^

While promising, the study’s findings should be interpreted considering several limitations. First, ICD-10 codes for specific causes of death were not available, and we used unnatural death as a proxy for suicide among individuals with a recent history of self-harm. This approach introduces uncertainty in model estimates. Misclassification could lead to prediction errors and may reduce the model’s specificity by obscuring distinctions between intentional and accidental deaths. This limitation underscores the urgent need for improved cause-of-death data and suicide surveillance to refine suicide prediction in LMICs. Nonetheless, unnatural death is a relevant and pragmatic endpoint in this context: it is closely linked to suicide among individuals presenting with self-harm,^6^ is more reliably recorded than suicide alone, and occurs more frequently, thereby enhancing statistical power and representing a meaningful target for prevention. Second, psychiatric and substance use disorders were identified from administrative claims data and exclude undiagnosed cases. While common mental disorders including depression (13%) and anxiety (16%) appear well-ascertained in this cohort,^6^ substance use disorders were recorded in less than 1% of beneficiaries, far below expected prevalence. This pattern likely reflects under-ascertainment of milder cases and possible reluctance of providers to code stigmatised conditions. We therefore interpret findings related to substance use disorders with caution. Finally, the model was developed and internally validated within a specific privately insured and HIV-programme-enrolled cohort. Compared with the broader South African population, this group has a higher socioeconomic status and greater access to healthcare. This limits generalisability to those relying on public-sector services who face greater psychosocial stressors, though evidence from South Africa suggests that key predictors of self-harm may be broadly consistent across socioeconomic groups.^29^

Future research should prioritise external validation in independent datasets, particularly in the public sector, to assess generalisability across broader populations with a history of self-harm.^28 30^ Prospective validation in clinical settings is also needed to confirm model calibration and real-world utility.^17 28^ In low-resource settings, embedding such validation within broader implementation research could enhance feasibility. Beyond validation, efforts should be made to explore the practical integration of prediction tools into routine care, for example, via web-based calculators, electronic health records, or mobile platforms, and evaluate feasibility, cost-effectiveness, acceptability, and effects on clinician decision-making and patient outcomes.^16^ Finally, prognostic models that estimate risk alone do not identify which interventions are most likely to benefit a given individual. A key next step is to develop individualised treatment rules that combine baseline risk with evidence on heterogeneity of treatment effects, with the aim of matching patients to interventions that maximise expected benefit. Achieving this will require randomised trial data with sufficient power to estimate effect modification across clinically relevant strata.

## Conclusions

The prediction models showed good discrimination, calibration and risk stratification, and effectively ranked individuals accessing private-sector care in South Africa by their probability of unnatural death after healthcare presentation for non-fatal self-harm and after discharge following a self-harm-related admission.

### Clinical implications

The models are intended to support clinical decision-making and guide the prioritisation of individuals with predicted high risk for targeted interventions, supporting more efficient allocation of mental healthcare capacity, which is often limited in low- and middle-income settings.

## Supplementary material

10.1136/bmjment-2025-302473 online supplemental file 1

## Data Availability

Data may be obtained from a third party and are not publicly available.
