# Supplementary material for: Risk of unnatural death following self-harm in South Africa: development and validation of multivariable prognostic models
Source: BMJ Ment Health. 2026 Jun 25;29(1):e302473. doi: 10.1136/bmjment-2025-302473 (PMC13311708; doi:10.1136/bmjment-2025-302473)
Supplement: online supplemental file 1 [file bmjment-29-1-s001.docx]

Risk of Unnatural Death Following Self-Harm in South Africa: Development and Validation of Multivariable Prognostic Models

Supplementary Figures, Tables, and Texts

**Table of Contents**

[Online supplemental text 1. Development and evaluation of the prediction models 2](#_Toc230263116)

[Online supplemental figure 1. Study inclusion flowchart 4](#_Toc230263117)

[Online supplemental figure 2. Adjusted hazard ratios for age as predictor of unnatural death 5](#_Toc230263118)

[Online supplemental figure 3. Adjusted hazard ratios for categorical predictors in the simplified models 6](#_Toc230263119)

[Online supplemental figure 4. LASSO cross-validation performance across penalty parameters 7](#_Toc230263120)

[Online supplemental figure 5. Calibration of the full presentation and discharge models 8](#_Toc230263121)

[Online supplemental figure 6. Calibration of the simplified presentation and discharge models 9](#_Toc230263122)

[Online supplemental figure 7. Risk stratification trade-off between the population classified as high risk and unnatural deaths captured for the full models 10](#_Toc230263123)

[Online supplemental figure 8. Risk stratification trade-off the between population classified high-risk and unnatural deaths captured for simplified models 11](#_Toc230263124)

[Online supplemental figure 9. Cumulative incidence of unnatural death after non-fatal self-harm in high- and low-risk groups at varying thresholds, compared with individuals without prior self-harm 12](#_Toc230263125)

[Online supplemental table 1. Overview of predictors, corresponding case definitions, and assessment periods 13](#_Toc230263126)

[Online supplemental table 2. Predictor contributions to the risk score across four prognostic models as percentage of variance in the linear predictor 14](#_Toc230263127)

[Online supplemental table 3. Cross-validated C-index for candidate models suggested by LASSO 15](#_Toc230263128)

# Online supplemental text 1. Development and evaluation of the prediction models

We developed four multivariable prognostic models for unnatural death after non-fatal self-harm: a full presentation model, a full discharge model, a simplified presentation model, and a simplified discharge model.

We used a two-stage approach for predictor selection. First, we screened binary predictors using the 2-year outcome of unnatural death. We retained predictors as candidates for main effects if at least two unnatural deaths occurred within 2 years in both exposure groups. We applied the same minimum-event rule to interactions between sex and mental disorders, psychiatric medication, and self-harm method, requiring at least two unnatural deaths within 2 years in each sex-by-exposure subgroup.

Second, we fitted penalized least absolute shrinkage and selection operator (LASSO) models using Cox regression with 20-fold cross-validation and the cross-validated Harrell’s concordance index (C-index) as the performance criterion. Age was modelled using a restricted cubic spline with 5 degrees of freedom, with internal knots at 25 and 35 years and boundary knots at 10 and 95 years. The spline coefficients for age were not penalized. All other candidate predictors were entered as categorical variables. The LASSO models included all main and interaction effects retained after screening. To preserve the outcome composition across folds, we created folds by stratified random sampling according to event status (unnatural death, natural or unknown death, or alive). We selected predictors at the value of λ_min_ that maximized the cross-validated C-index.

After penalized selection, we applied two additional rules. First, if an interaction term was selected, we included the corresponding main effects. Second, if any level of a multi-level categorical predictor was selected, we included all levels of that predictor.

Next, we refitted the selected predictors in unpenalized Fine-Gray subdistribution hazards models to estimate the cumulative incidence of unnatural death, treating natural deaths and deaths from unknown causes as competing events. We used the resulting models to estimate individual 2-year predicted risks of unnatural death. All models were developed and evaluated in the same cohort. We used bootstrap internal validation to quantify optimism.

We assessed model performance in terms of discrimination, calibration, and risk stratification. Discrimination was quantified using the C-index for competing risks. Calibration was assessed graphically and numerically. For graphical calibration, we grouped individuals into deciles of predicted risk and plotted the observed 1-, 2-, and 3-year cumulative incidence of unnatural death, estimated using the Aalen-Johansen estimator, against the mean predicted cumulative incidence within each decile. For numerical calibration, we estimated 2-year calibration intercepts and slopes on the log-log scale using inverse probability of censoring weighting to account for censoring before 2 years.

We assessed risk stratification by ranking individuals according to their predicted 2-year risk and quantifying the proportion of all unnatural deaths occurring within 1, 2, and 3 years that were captured within high-risk groups defined by increasing thresholds of predicted 2-year risk. In the risk stratification analyses, we evaluated thresholds corresponding to the top 10% to top 70% of the predicted risk distribution.

We report the apparent performance and optimism-corrected performance for discrimination, calibration intercept and slope, and risk stratification, assessed as the proportion of unnatural deaths captured within high-risk groups. Apparent performance refers to model performance evaluated in the same dataset used for model development. We used bootstrap internal validation with 1000 resamples to quantify optimism in these performance measures. In each bootstrap resample, we repeated the full model-development procedure, including predictor and interaction screening based on minimum-event rules, penalized LASSO selection using 20-fold cross-validation, enforcement of hierarchical selection rules, model selection based on λmin, and refitting of the selected Fine-Gray model. For each bootstrap model, we estimated apparent performance in the bootstrap sample and test performance in the original dataset, and defined optimism as the difference between these two estimates. We obtained optimism-corrected performance metrics by subtracting the mean bootstrap optimism from the corresponding apparent estimates in the original dataset.

We ranked predictors by the proportion of variance in the linear predictor accounted for by each predictor term. We calculated this proportion as the variance in the term-specific component of the risk score divided by the total variance of the full risk score and multiplied by 100. We treated multi-parameter terms, such as spline-transformed age and multi-level categorical predictors, as single predictors. Interaction terms were reported separately.

Data management was performed using Stata version 18 (StataCorp, College Station, TX, USA) and R version 4.3.1 (R Foundation for Statistical Computing, Vienna, Austria), and statistical analyses were performed in R.

# Online supplemental figure 1. Study inclusion flowchart

**
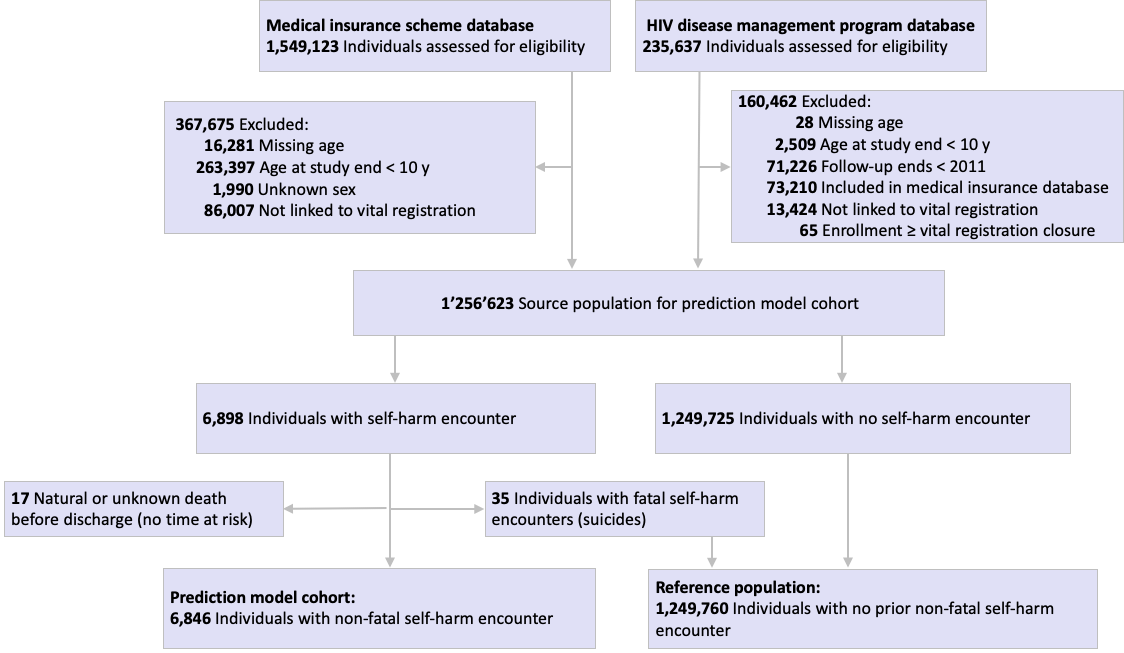
**

# Online supplemental figure 2. Adjusted hazard ratios for age as predictor of unnatural death

Estimates are subdistribution hazard ratios with 95% confidence intervals for the association between age and unnatural death after non-fatal self-harm, modelled as continuous predictor with restricted cubic splines. Panel A shows the full presentation model, Panel B the full discharge model, Panel C the simplified presentation model, and Panel D the simplified discharge model. Hazard ratios are adjusted for all variables included in the respective model.


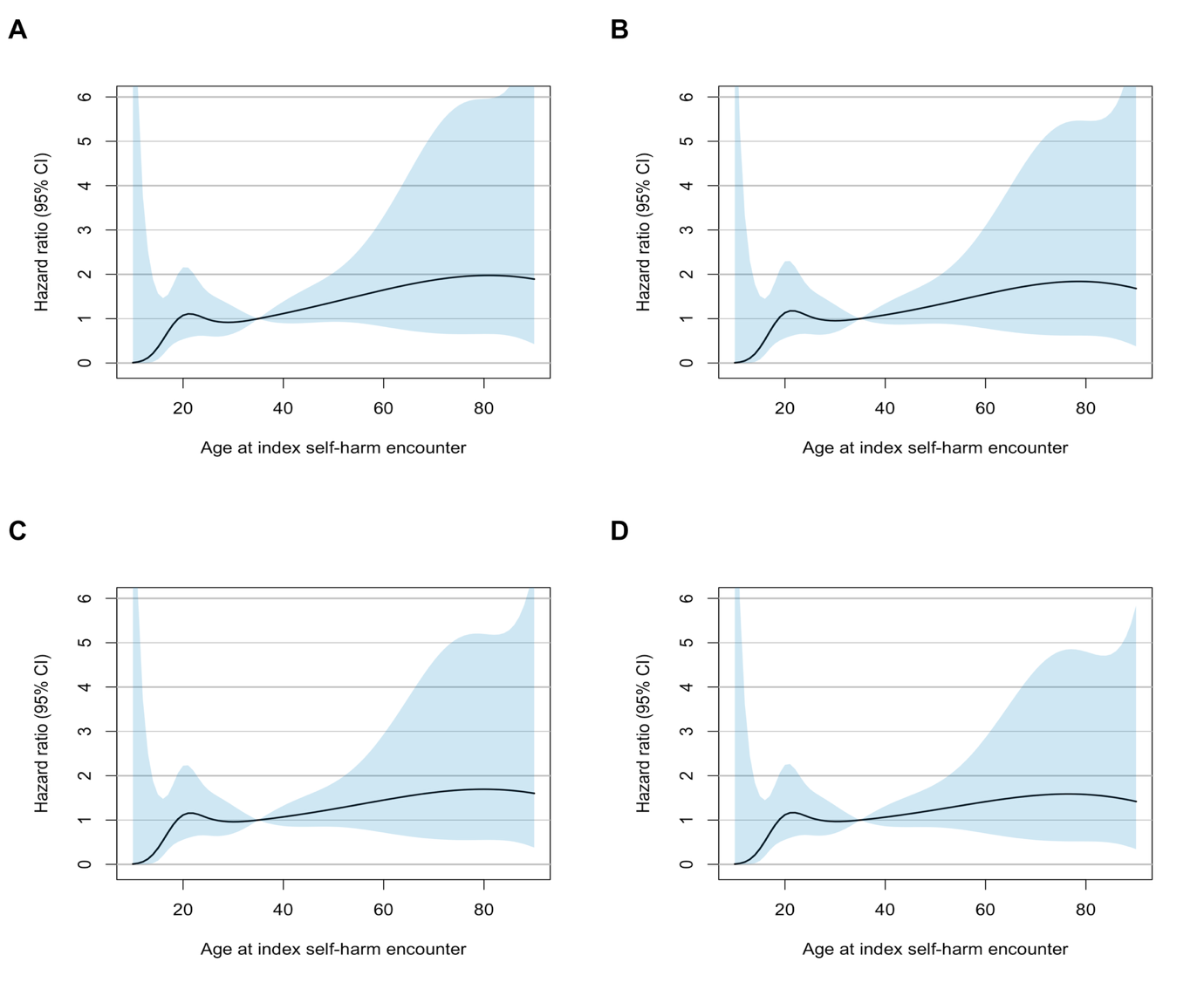


# Online supplemental figure 3. Adjusted hazard ratios for categorical predictors in the simplified models

Estimates are subdistribution hazard ratios with 95% confidence intervals for unnatural death after non-fatal self-harm. Panel A shows results for categorical predictors included in the best-performing simplified multivariable presentation model. Panel B shows results for categorical predictors included in the best-performing simplified discharge model. For variables included in interaction terms, the reported hazard ratios refer to the reference category of the interacting variable. Both models also included age, modelled as continuous predictor with restricted cubic splines. The association for age is shown in online supplemental figure 2.


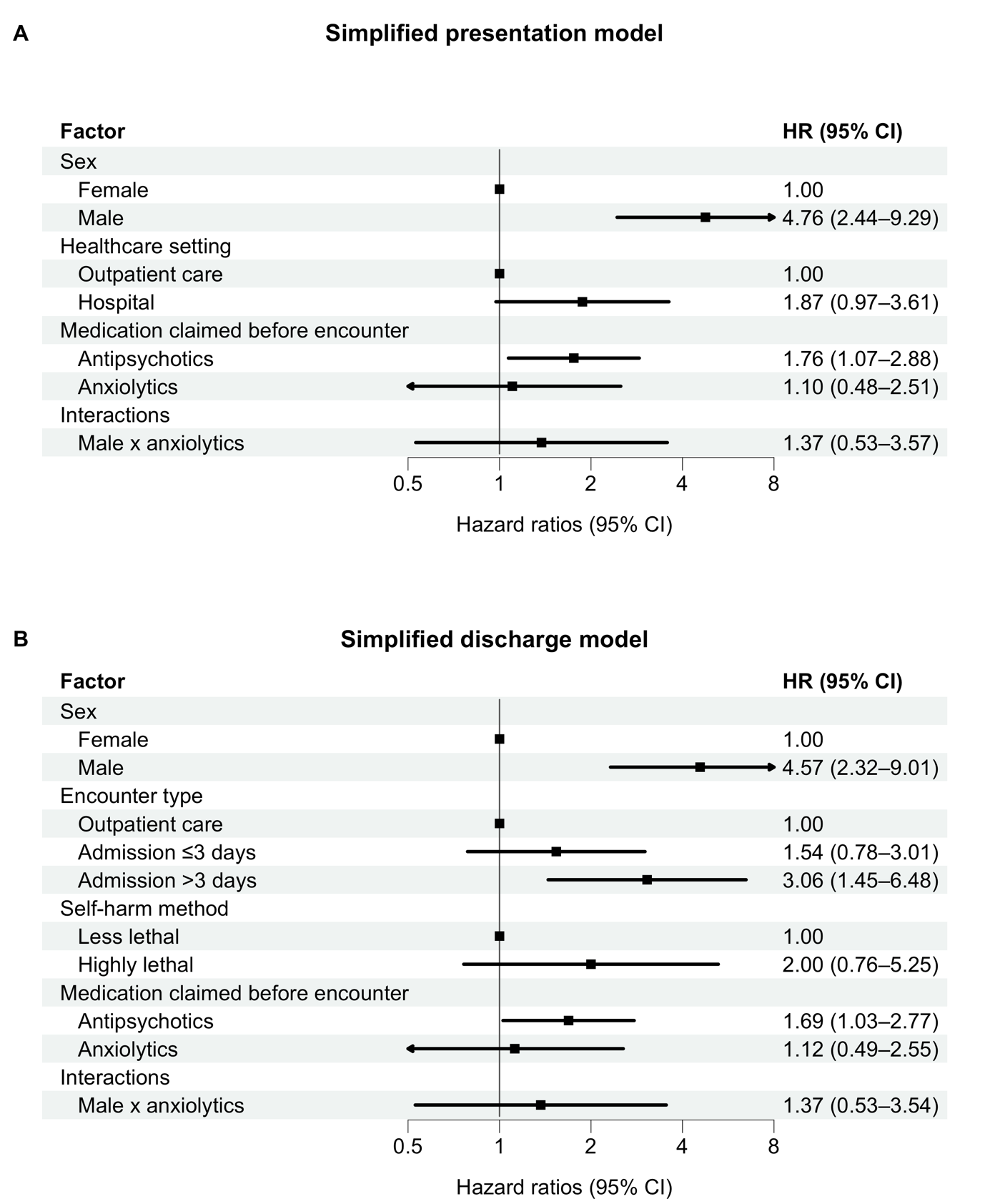


# Online supplemental figure 4. LASSO cross-validation performance across penalty parameters

Cross-validated concordance indices (C-index) from 20-fold cross-validation are shown across a range of LASSO penalty parameters (λ) for the full presentation model (A), full discharge model (B), simplified presentation model (C), and simplified discharge model (D). The dashed grey line indicates the penalty parameter with the highest mean C-index (λmin). The dashed lines indicate the penalty parameter with the highest mean C-index (λmin) and the penalty parameter corresponding to a one standard deviation decrease in mean C-index relative to λmin (λ1se). The top of each panel shows the number of selected non-zero coefficients (panel size) at each λ value.


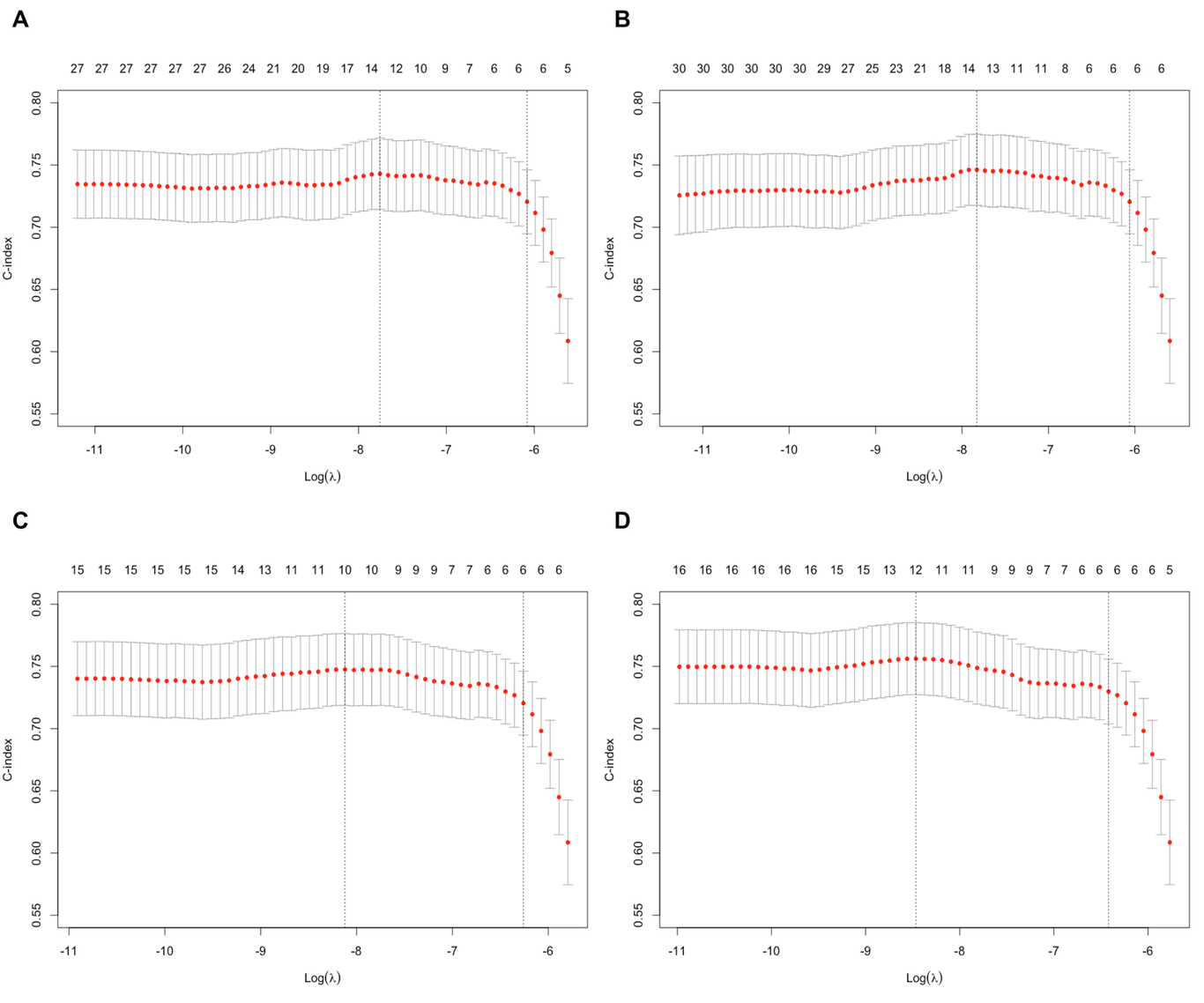


# Online supplemental figure 5. Calibration of the full presentation and discharge models

Apparent observed 1-year, 2-year, and 3-year risks of unnatural death, with 95% confidence intervals (error bars), are plotted against the mean predicted risks within deciles of predicted risk in the same dataset used for model development. The diagonal line represents perfect agreement between predicted and observed risks.


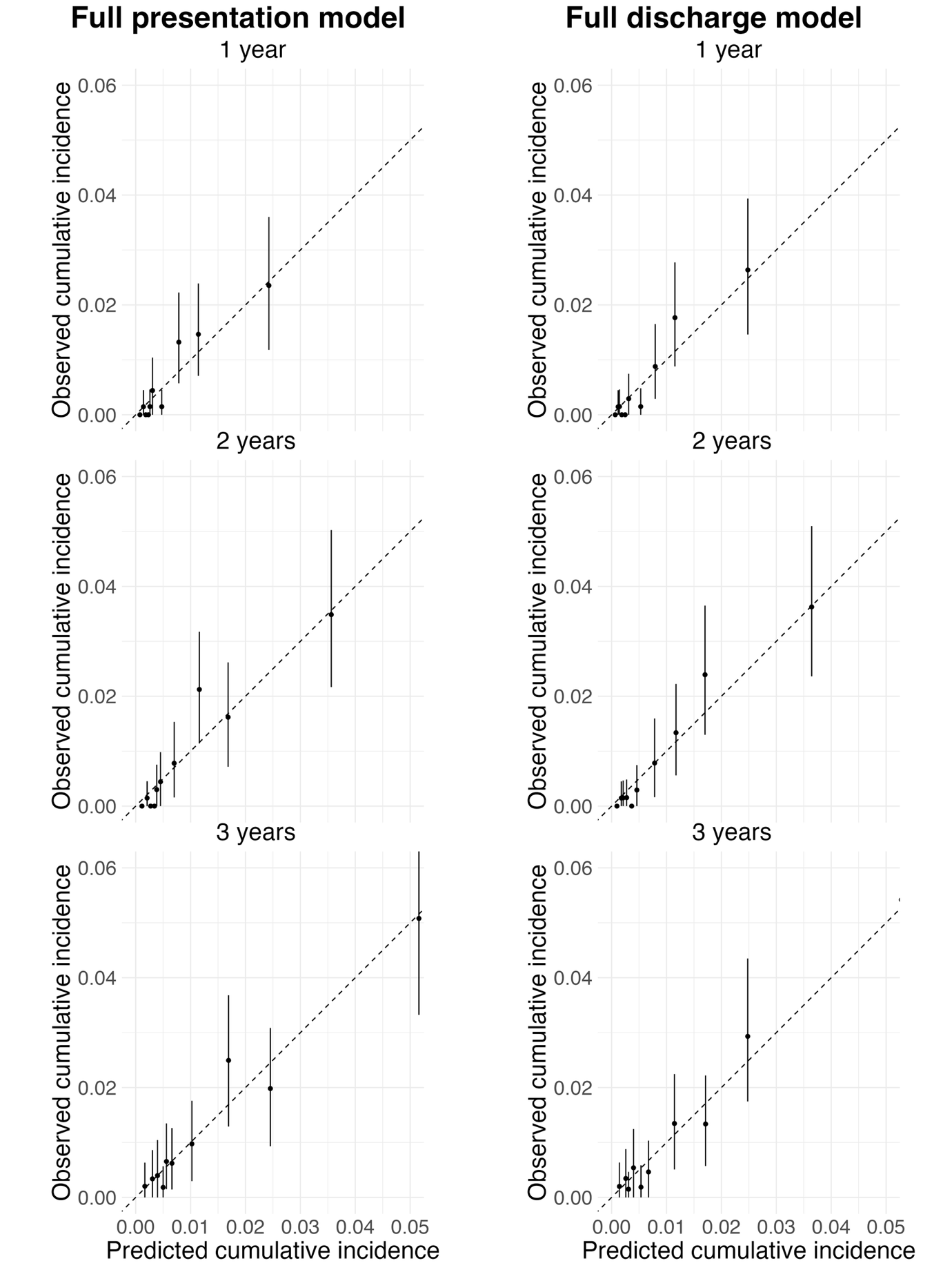


# Online supplemental figure 6. Calibration of the simplified presentation and discharge models

Apparent observed 1-year, 2-year, and 3-year risks of unnatural death, with 95% confidence intervals (error bars), are plotted against the mean predicted risks within deciles of predicted risk in the same dataset used for model development. The diagonal line represents perfect agreement between predicted and observed risks.


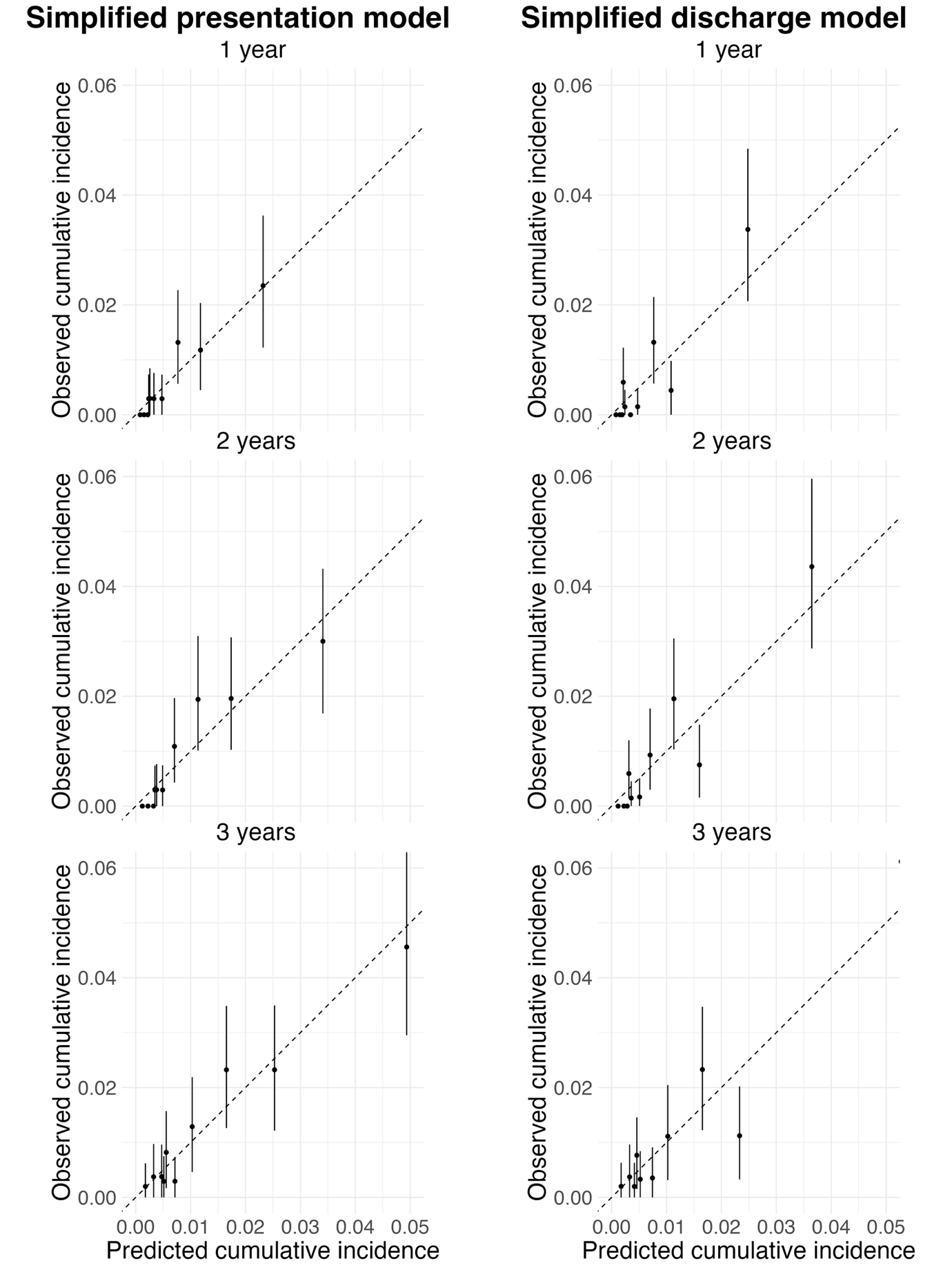


# Online supplemental figure 7. Risk stratification trade-off between the population classified as high risk and unnatural deaths captured for the full models

The figure plots the apparent proportion of all unnatural deaths occurring within 1, 2, and 3 years that fall within high-risk groups, defined by increasing thresholds of predicted risk, against the corresponding proportion of individuals classified as high risk. Panel A shows results for the best-performing full presentation model. Panel B shows results for the best-performing full discharge model. Optimism-corrected estimates at selected high-risk thresholds are presented in Table 2.


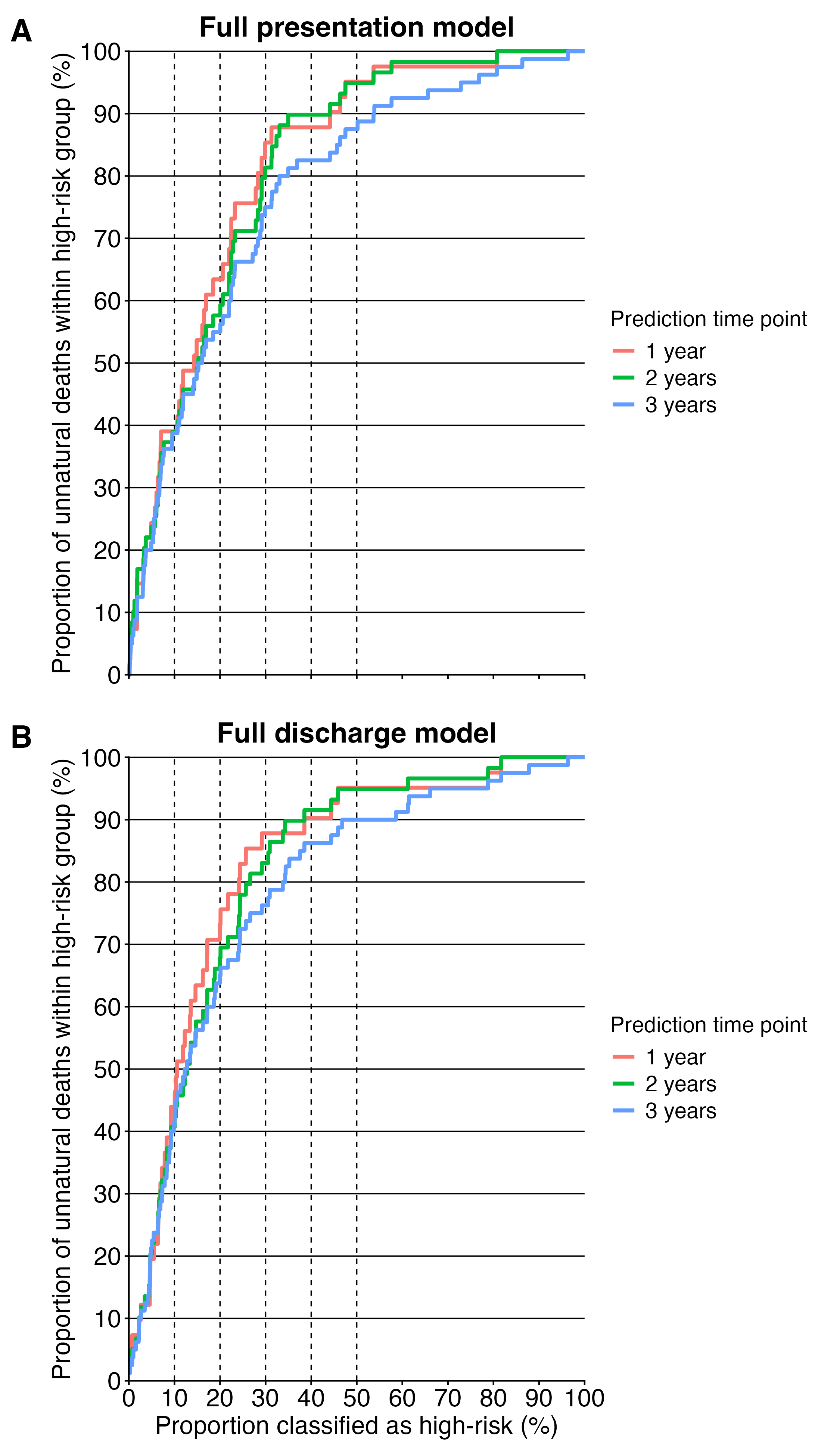


# Online supplemental figure 8. Risk stratification trade-off the between population classified high-risk and unnatural deaths captured for simplified models

The figure plots the apparent proportion of all unnatural deaths occurring within 1, 2, and 3 years that fall within high-risk groups, defined by increasing thresholds of predicted risk, against the corresponding proportion of individuals classified as high risk. Panel A shows results for the best-performing simplified presentation model. Panel B shows results for the best-performing simplified discharge model. Optimism-corrected estimates at selected high-risk thresholds are presented in Table 2.


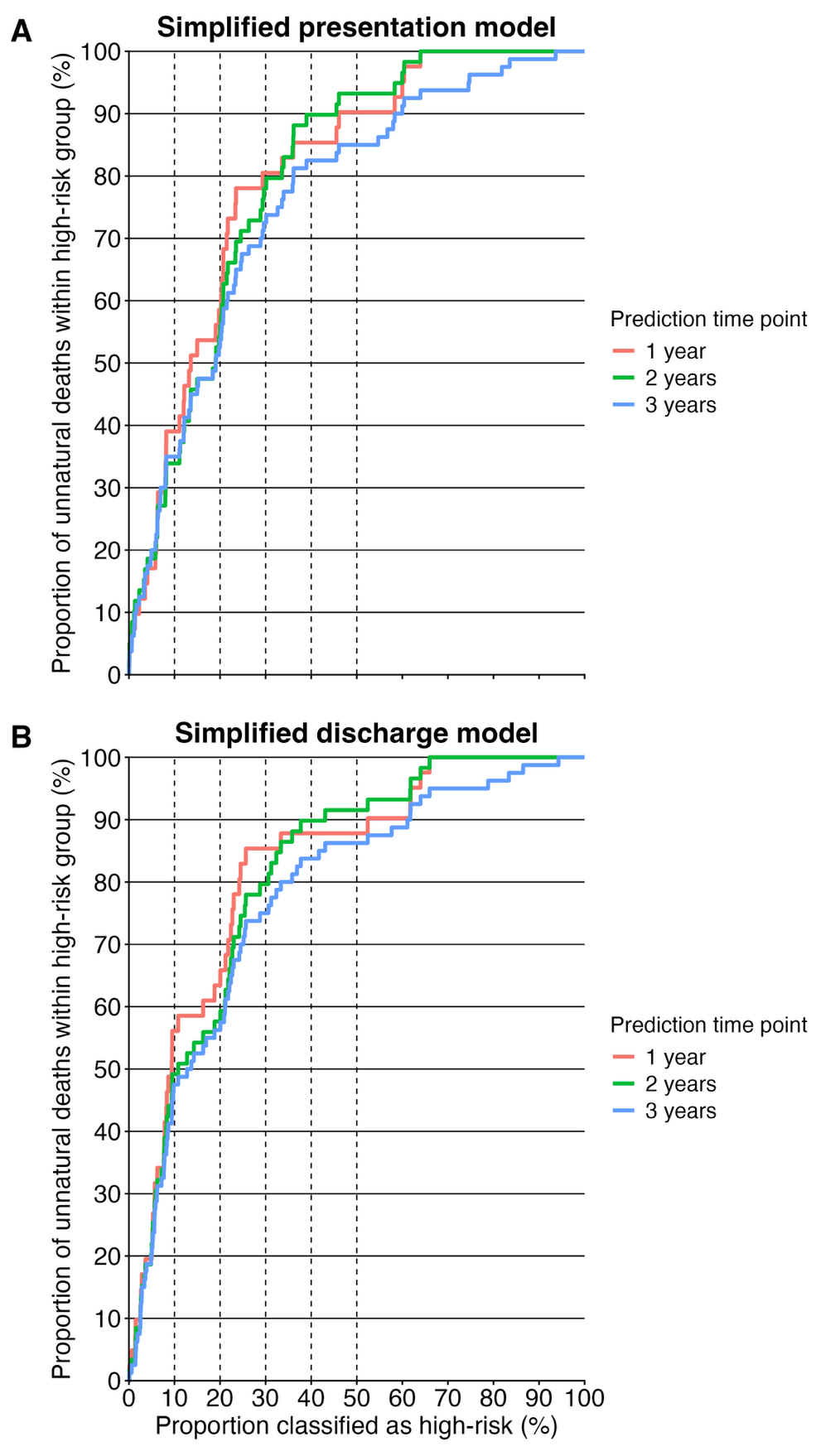


# Online supplemental figure 9. Cumulative incidence of unnatural death after non-fatal self-harm in high- and low-risk groups at varying thresholds, compared with individuals without prior self-harm

The figure shows cumulative incidence of unnatural death with 95% confidence intervals after non-fatal self-harm (NFSH) among high-risk groups, defined as the top 10% (A) and top 40% (B) by predicted risk, compared with the corresponding lower-risk groups (bottom 90% in A, and bottom 60% in B) and individuals without a prior non-fatal self-harm encounter. Predictions are based on the discharge model.


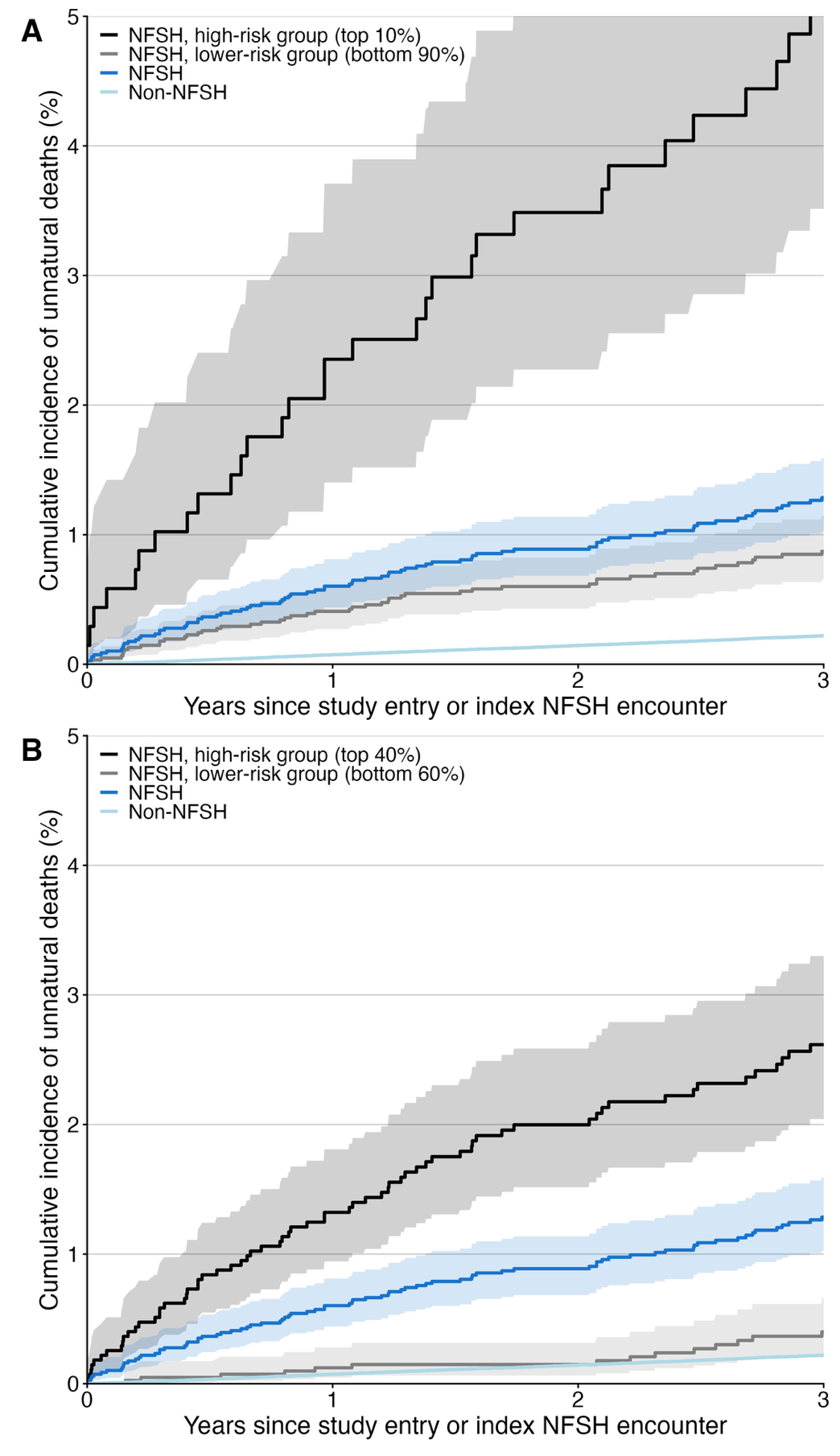


# Online supplemental table 1. Overview of predictors, corresponding case definitions, and assessment periods

| **Predictor** | **Definition** | **Assessment point or window** |
| --- | --- | --- |
| **Demographic characteristics** |  |  |
| Sex | male, female | at self-harm encounter date |
| Age | continuous | at self-harm encounter date |
| **Encounter characteristics** |  |  |
| Health care setting | outpatient care, hospital setting | at self-harm encounter date |
| Encounter type | outpatient care, admission ≤3 days, admission >3 days | at self-harm encounter date |
| Self-harm method | highly lethal or less lethal method at index encounter.  Highly lethal methods included: self-harm by drowning and submersion (ICD10 code X71), firearms or explosives (X72–X75), hanging, strangulation and suffocation (X70), jumping from height or in front of a moving object (X80-X81), and self-poisoning by gases (X67).  Less lethal methods included: self-poisoning other than gas (X60–X66, X68–X69), sharp object (X78), blunt object (X79), smoke, fire and flames (X76), steam, hot vapours and hot objects (X77), crashing of a motor vehicle (X82), and other or unspecified means (X83–X84). | at self-harm encounter date |
| **Mental disorder diagnoses** |  |  |
| Any mental disorder | F00-F99 | - before self-harm encounter date (history)  - up to the discharge date for an admission related to the self-harm encounter (history and current diagnoses) |
| Organic mental disorders | F00-F09 |  |
| Substance use disorders | F10-F16, F18-F19 |  |
| Alcohol use disorders | F10 |  |
| Drug use disorders | F11–F16, F18–F19 |  |
| Psychotic disorders | F20–F29 |  |
| Mood disorders | F30–F39 |  |
| Bipolar disorder | F31 |  |
| Major depression | F32, F33, F34.1 |  |
| Anxiety disorders | F40–F48 |  |
| PTSD | F43.1 |  |
| Other anxiety disorders | F40–F42, F43.0, F43.2–F48 |  |
| Personality disorders | F60–F69 |  |
| Other mental disorders | F50–F59, F70–F99 |  |
| **Psychiatric medication** |  |  |
| Antipsychotics | N05A | history |
| Anxiolytics | N05B | history |
| Antidepressants | N06A | history |
| Drugs with anti-suicidal effect | Lithium (N05AN01), Clozapine (N05AH02) | history |
| **Physical illnesses** |  |  |
| HIV | positive or negative.  Positive if the individual has ever received an HIV-related diagnosis (B20-B24, Z21, R75, O98.7), has ever used antiretroviral medication for HIV treatment (J05AE, J05AF, J05AG, J05AJ, J05AR), excluding medications used for pre- or post-exposure prophylaxis (J05AR03, J05AF13, J05AF09, J05AF05), has ever received a positive HIV test or undergone an HIV viral load test or CD4 cell count test, or has ever been registered in the HIV disease management program.  Negative if none of the above criteria have been met. | at self-harm encounter date |

Online supplemental table 2. Predictor contributions to the risk score across four prognostic models as percentage of variance in the linear predictor

| **Predictor** | **Full presentation model** | **Full discharge model** | **Simplified presentation model** | **Simplified discharge model** |
| --- | --- | --- | --- | --- |
| Sex | 37.3 | 25.2 | 51.3 | 46.7 |
| Age | 22.6 | 17.5 | 23.3 | 21.5 |
| Encounter type |  | 8.2 |  | 10.0 |
| Healthcare setting | 5.4 |  | 6.3 |  |
| Self-harm method |  |  |  | 0.9 |
| Antipsychotics | 1.6 | 1.6 | 5.2 | 4.3 |
| Anxiolytics | 0.7 | 0.1 | 0.2 | 0.3 |
| Drug use disorder diagnosis | 1.2 |  |  |  |
| Bipolar disorder diagnosis | 1.3 | 2.0 |  |  |
| Depression diagnosis | 5.8 | 11.8 |  |  |
| Sex × Anxiolytics | 0.1 | 0.7 | 0.9 | 0.8 |
| Sex × Depression diagnosis | 4.6 | 7.5 |  |  |

Values are percentages of the variance in the linear predictor attributable to each predictor block. Predictor contributions were calculated separately for each model using the fitted linear predictor, with spline terms for age and categories for encounter type grouped into blocks. Interaction terms are shown as separate blocks where present. Empty cells indicates that the predictor was not included in the corresponding model.

Online supplemental table 3. Cross-validated C-index for candidate models suggested by LASSO

| **Model** | **Apparent C-index** | **Optimism-corrected C-index** | **Apparent calibration intercept** | **Optimism-corrected calibration intercept** | **Apparent calibration slope** | **Optimism-corrected calibration slope** |
| --- | --- | --- | --- | --- | --- | --- |
| Full presentation model | 0.7847313 | 0.7386955 | -0.02977077 | -0.01927400 | 1.140635 | 0.8863877 |
| Full discharge model | 0.7987907 | 0.7508931 | -0.03156817 | -0.02269987 | 1.100544 | 0.8407287 |
| Simplified presentation model | 0.7719023 | 0.7381823 | -0.03013398 | -0.01342119 | 1.119142 | 0.9166444 |
| Simplified discharge model | 0.7903550 | 0.7563788 | -0.03256425 | -0.01644947 | 1.096591 | 0.9005447 |

Calibration was assessed at the 2-year prediction horizon. Calibration intercept values close to 0 indicate little evidence of systematic overprediction or underprediction of absolute risk. Calibration slope values close to 1 indicate good agreement between predicted and observed risk gradients. Values below 1 suggest some overfitting, with predictions that are too extreme. Optimism-corrected estimates were obtained by bootstrap internal validation of the full modelling pipeline, including candidate predictor screening, LASSO-based selection, and refitting of the selected Fine-Gray model.
